# Direct Evidence for the Co-Expression of URP and GnRH in a Sub-Population of Rat Hypothalamic Neurones: Anatomical and Functional Correlation

**DOI:** 10.1371/journal.pone.0026611

**Published:** 2011-10-24

**Authors:** Johann-Günther Egginger, Caroline Parmentier, Ghislaine Garrel, Joëlle Cohen-Tannoudji, Alain Camus, André Calas, Hélène Hardin-Pouzet, Valérie Grange-Messent

**Affiliations:** 1 Laboratoire de Neurobiologie des Signaux Intercellulaires, CNRS UMR 7101-Université Pierre et Marie Curie, Paris, France; 2 Laboratoire de Physiopathologie des Maladies du Système Nerveux Central, INSERM U952/CNRS UMR 7224-Université Pierre et Marie Curie, Paris, France; 3 Physiologie de l'axe gonadotrope, Unité de Biologie Fonctionnelle et Adaptative, CNRS EAC 4413- Université Denis Diderot-Paris 7, Paris, France; 4 Laboratoire de Neurobiologie des Signaux Intercellulaires, CNRS UMR 7101-Université Pierre et Marie Curie, Paris, France; 5 Interdisciplinary Institute for Neuroscience (IINS), CNRS UMR 5297- Université Bordeaux-Segalen, Bordeaux, France; Université Pierre et Marie Curie, France

## Abstract

Urotensin-II-related peptide (URP) is an eight amino-acid neuropeptide recently isolated from rat brain and considered as the endogenous ligand for the GPR14 receptor. Using single and double immunohistochemical labelling, *in situ* hybridization and ultrastructural immunocytochemistry, we explored the cellular and subcellular localization of URP in the male rat brain. URP peptide was detected in numerous varicose fibres of the median eminence (ME) and *organum vasculosum laminae terminalis* (OVLT) as well as in neuronal cell bodies of the medial septal nucleus and diagonal band of Broca where corresponding mRNA were also detected. Combining *in situ* hybridization with immunohistochemistry, we showed that cell bodies of the rat anterior hypothalamus contained both URP mRNA and GnRH peptide. In addition, double ultrastructural immunodetection of URP and GnRH peptides clearly revealed, in the median eminence, the co-localization of both peptides in the same neuronal processes in the vicinity of fenestrated portal vessels. This remarkable cellular and subcellular distribution led us to test the effect of URP on the GnRH-induced gonadotrophins release in the anterior pituitary, and to discuss its putative role at the level of the median eminence.

## Introduction

In vertebrate, sexual maturation and reproduction are governed by the gonadotrophin-releasing hormone (GnRH) system which regulates the function of gonads [1]–[3]. In rodents, GnRH peptide is expressed in neuroendocrine cell bodies mainly located in the hypothalamic preoptic area, and is released into the hypophyseal portal circulation in the median eminence for delivery to the anterior pituitary. At the adenohypophysis, GnRH modulates the synthesis and secretion of gonadotrophins LH and FSH, which stimulate gonadal steroid secretion and gametogenesis. Regulation of GnRH neurones activity is complex and is controlled by several excitatory and inhibitory transsynaptic inputs [1], [3]–[7]. These synaptic regulatory mechanisms lead to a function-related structural plasticity of the GnRH system involving neuronal-glial-endothelial interactions (see for review [8]), so allowing an answer adapted by the system to the physiological situations. Neuroendocrine GnRH neurones are also known to present a various pattern of chemical phenotype, expressing some neuropeptides such as galanin [9], [10], cholecystokinin and neurotensin [11]. These co-expressed neuropeptides could act on the neuroendocrine activity of the GnRH neurones by modulating GnRH release at the nerve endings and/or godatropins release at the anterior pituitary. We have showed in a preliminary study that Urotensin II-related peptide (URP) and its corresponding mRNA were also present within GnRH neurones in male mouse hypothalamus proposing this peptide, for the first time, as a novel hypothalamus neuroendocrine peptide [12]. URP was first isolated from rat brain extracts and is considered as the endogenous ligand of the orphan receptor GPR14 [13]–[15]. Primary structure of URP is very close to that of the neuropeptide Urotensin II which is involved in several biological activities (see for reviews [16]–[18]). Actually physiological role of URP in the central nervous system remains unknown. Interest of UII function in the mammalian central nervous system has only emerged very recently with the discovery of its potential role in sleep or behaviour (see for reviews in [19], [20]).

Our present data demonstrate that URP and its mRNA are also expressed in a subpopulation of GnRH neurones in the male rat hypothalamus using immunohistochemistry combining with *in situ* hybridization. To gain further information, these data are extended by electron microscopic double immunocytochemistry experiments which exhibit the co-localization of both peptides URP and GnRH in the same nerve processes of the median eminence in the vicinity of fenestrated portal vessels. Taken together, these results prompted us to examine if URP may affect basal or GnRH-induced LH and FSH release in primary culture of anterior pituitary cells.

## Materials and Methods

### Ethics statement

All efforts were made to minimise animal suffering and the number of animals used. They were anaesthetised and other respects treated in accordance with the European Communities Council Directive of 24th November 1996 (86/609/EEC). All animal experiments were approved by the local committee on animal welfare, the French Department of Veterinary Service, which delivered to VGM and to HHP the authorizations 75-31 and 75-1650 respectively,to experiment on alive animals.

### Animals

Twenty-two adult male Sprague Dawley rats aged from six to seven weeks (Janvier, Saint-Berthevin, France) and weighing 200–250 g were housed under constant temperature (20±2°C) and lighting (light on from 07.00 to 19.00) regimens. They had free access to standard rat food and tap drinking water.

### Immunohistochemistry

Rats (n = 6) were anaesthetised with intraperitoneal injection of sodium pentobarbital (25 mg/kg; Sanofi Santé Animale, Libourne, France) and transcardially perfused with saline solution and then with 4% paraformaldehyde in 0.1 M phosphate buffer (PB), pH 7.4. Brains were immediately removed and post-fixed in the same fixative for 2 h at 4°C and cryoprotected in a 20% sucrose/0.05 M PB solution overnight. Frontal and horizontal 20 µm sections including hypothalamus were cut using a cryostat and used immediately for immunofluorescence or peroxidase immunohistochemistry.

#### URP and URP/GnRH fluorescent immunodetection

Sections from 3 rat brains were first incubated with 5% bovine serum albumin (BSA) with 0.1% Triton X-100 in 0.05 M phosphate buffered saline (PBS) for 1 h in a humid chamber at room temperature (RT). Then sections were incubated with rabbit polyclonal IgG raised against rat URP (Phoenix Pharmaceuticals Inc., Belmont CA) at a dilution of 1∶1000 for simple immunofluorescence, or with a mix containing URP-antiserum (1∶1000) and mouse monoclonal GnRH-antibodies (1∶1000; specific for mammalian GnRH C-terminal pentapeptide; Sternberger Monoclonals Incorporated, Lutherville, Maryland, USA) for double immunofluorescence, in the PBS/Triton/BSA solution for 48 h at 4°C. After washes, the immune complex URP antigen-first antibody for simple immunofluorescence was detected using biotinylated goat anti-rabbit IgG (1∶200 dilution in BSA/Triton/PBS; Vector Laboratories, Burlingame, USA) for 2 h at RT followed by washes in PBS and incubation with Cy3 Conjugated- Streptavidin (1∶200 dilution in PBS; Sigma, St Louis, MO, USA) for 1 h at RT. For double immunofluorescence, immune complexes URP antigen-first antibody and GnRH antigen-first antibody were detected using Cy3-conjugated sheep anti-rabbit IgG (1∶200 dilution in PBS; Sigma) and FITC-conjugated horse anti-mouse IgG (1∶200 dilution in PBS; Vector) for 1 h at RT for 2 h at RT. All sections were examined with a *Leica DMRB* fluorescence microscope. Some sections were also examined under a *Leica TCS-SP* confocal microscope.

#### URP peroxidase immunodetection

Three rat brains were used for these experiments and treated as described above. After incubation with primary URP antibody and washes, sections were incubated with biotinylated anti-rabbit IgG at a dilution of 1∶200 in the PBS/Triton/BSA solution for 2 h at RT. Following washes in PBS, sections were incubated with 0.006% H_2_O_2_ (Sigma) for 15 min at RT in order to remove endogenous peroxidase activity and then incubated for 1 h at RT with peroxidase-labelled ABC kit (1∶100 avidin and 1∶100 biotin; Vector Laboratories, Burlingam, USA) in PBS. HRP peroxidase activity was revealed by incubating the sections in 0.05 M Tris buffer, pH 7.5, containing 0.05% DAB (3,3′-diaminobenzidine; Sigma) and 0.006% H_2_O_2_ (Sigma) for 15 min at RT. Finally, sections were observed using a *Leica DMRB* light microscope.

#### Immunohistochemistry controls

To study the specificity of the URP-immunoreaction, the following controls were performed: 1) substitution of both the URP-antiserum and GnRH-antiserum with 0.05 M PBS, 2) pre-incubation of the URP-antiserum (diluted 1∶1000) with rat URP synthetic peptide (up to 10^−4^ M; lot# 420956, Phoenix Pharmaceuticals Inc.), 3) pre-incubation of the URP-antiserum (diluted 1∶1000) with mammalian GnRH 1–10 peptide (up to 10^−7^ M, in wide excess with regard to the anti-URP IgG; Sigma), and GnRH-antiserum (diluted 1∶1000) with rat URP synthetic peptide (up to 10^−4^ M). Absence of homology between rat URP peptidic sequence and other hypothalamic peptide sequences such as delta sleep-inducing peptide (DSIP) was verified using Basic Local Alignment Search Tool, BLAST®, to exclude cross reaction.

### Electron microscope immunocytochemistry

#### Simple immunodetection of URP

Rats (n = 4) were anaesthetised as described above and perfused with a saline solution and then with a solution containing 0.2% glutaraldehyde, 4% paraformaldehyde in 0.1 M PB, pH 7.4 and 1.2% saturated picric acid. Brains were immediately removed, post-fixed in the same fixative solution for 4 h at 4°C and cut using a vibratome. Frontal brain sections (40 µm) including median eminence (ME) were processed using the same procedure as for peroxidase immunohistochemistry described above.

#### Double immunodetection of URP and GnRH

Rats (n = 4) were used and processed as described above. Frontal sections (40 µm) were treated by incubation in 0.05 M PBS containing 1% BSA for 30 min. This pre-incubation was followed by incubation overnight at RT with primary antibodies, rabbit polyclonal anti-URP antibody (Phoenix Pharmaceuticals Inc., Belmont, USA) and mouse monoclonal anti-GnRH antibody (Sternberger Monoclonals Incorporated, Lutherville, Maryland, USA) diluted 1∶500 and 1∶1000 respectively in 0.05 M PBS-1% BSA. After washes in PBS/BSA, URP-anti-URP antibody immune complex was detected by using biotine conjugated-anti-rabbit IgG diluted 1∶200 in 0.05 M PBS-1% BSA, for 2 h at RT. Following washes in PBS, sections were incubated for 1 h at RT with peroxidase-labelled ABC kit (1∶100 avidin and 1∶100 biotin, Vector Laboratories, Burlingam, USA). The peroxidase activity was revealed using a solution containing 0.05% DAB and 0.006% H_2_O_2_ in 0.05 M Tris buffer, pH 7.6. Reaction was stopped by immersion in Tris buffer and sections were transferred in PBS. This molecular scaffolding was fixed by 4% paraformaldehyde in 0.1 M PBS overnight at 4°C. Then, sections were rinsed in 0.05 M PBS before being pre-incubated in PBS-1% BSA solution for 30 min at RT. GnRH-anti GnRH immune complex was detected by incubation for 4 h at RT in a solution containing 0.8 nm colloidal gold-labelled goat-anti-mouse IgG/M antibody (Biovalley, Marne la Vallée, France) diluted 1∶100 in PBS-1% BSA. For intensification of the gold particles by silver, a commercial silver enhancement kit (Sigma) was used according to the recommendations of the company.

#### Embedding

At the end of immunodetection procedures, labelled sections were washed in 0.1 M sodium cacodylate buffer, post-fixed in 2% OsO_4_/0.2 M sodium cacodylate buffer (v/v) for 1 h, dehydrated through an alcohol ascending series and embedded in epon resin (Polysciences Inc., Warrington, PA) polymerized at 60°C during 48 h. Labelled areas were sectioned using a LKB ultramicrotome. Ultrathin sections were contrasted with uranyl salts, and then observed with a transmission *JEOL 100-CX II* electron microscope.

#### Immunocytochemistry controls

Control sections represented sections in which the first antibody either against URP or GnRH was omitted during immunostaining procedures. All control sections did not show non-specific staining even at the electron microscopic level.

### In situ hybridization

#### Probes

Three oligonucleotides probes, complementary to rat URP-mRNA coding sequence (*GenBank* accession NM_198133), were used in this study:

5′-AAATACCTCCGTTTCTCACGACTCGGCAGGTTACG-3′

5′-GGGACCAAAGGTCTCCGGGCGGGTACGACCACATC-3′

5′-GAGAGACGAAACCTGAGGACCGAAACAACAGACAC-3′

The specificity of the probe for the corresponding rat mRNA was verified by checking the DNA Database of Japan. These probes were labelled at the 3′ end with digoxigenin-11-dUTP (DIG-dUTP) using the commercial DIG oligonucleotide tailing kit (Roche, Mannheim, Germany). Absence of homology between rat URP-mRNA sequence and sequences of mRNA coding other hypothalamic peptides such as Urotensine II and delta sleep-inducing peptide (DSIP) was confirmed using, so excluding any crossed reaction.

#### URP-mRNA in situ hybridization

Four rats were used for simple *in situ* hybridization (ISH). Brain tissues were fixed and collected as described above for immunohistochemistry. Serial coronal frozen sections (40 µm) were cut and collected in two alternate series of consecutive sections in sterile culture dishes containing PBS. One batch of sections was used for peroxidase immunohistochemistry (see above) and the second one was used for in situ hybridization (ISH). All hybridization steps were carried out on free-floating sections in sterile culture dishes. The sections were rinsed several times in sterile PBS. For ISH, sections were incubated for 1 h at 37°C in a pre-hybridization solution containing 20% of standard saline citrate (SSC) [20× SSC = 3 M NaCl, 0.3 M sodium citrate; Sigma], 1% of 1× Denhardt's solution (Sigma), 5% of salmon testes DNA (9.9 mg/ml; Sigma), 1% dextran sulphate (Sigma) and double distilled water. Then, sections were incubated for 48 h at 37°C with 2 nmol/l of each digoxigenin-labelled URP probe. Probes were diluted in an hybridization solution containing 50% deionised formamide (Sigma), 20% of ISH solution [175 mg/ml NaCl, 7.5 mg/ml EDTA, 10 mg/ml *N*-lauroyl-sarcosine (Sigma), 2.5 mg/ml disodium-pyrophosphate (Sigma), 2.5 mg/ml tetrasodium-pyrophosphate (Sigma), 0,4 M Tris base pH 7.5], 5% of salmon testes DNA (9.9 mg/ml; Sigma), 1% dextran sulphate (Sigma) and double distilled water. Sections were then washed (42°C, 2× SSC; 1× SSC; 0.5× SSC; room temperature, 0.1× SSC) and processed for immunodetection of digoxigenin [21] with anti-DIG antibody conjugated to alkaline phosphatase (Roche, France). The NBT/BCIP precipitate was observed under a light microscope.

#### In situ hybridization controls

Control experiments were performed by hybridization of a few sections with sense probes and with an excess (50×) of unlabelled oligonucleotides antisense probe, in order to control the specificity of the hybridization signal.

### URP-mRNA *in situ* hybridization combined with GnRH immunohistochemistry

Rats (n = 4) were anaesthetised and perfused using the same procedure as described above. Brains were immediately removed and post-fixed in the same fixative for 2 h at 4°C and frontal 40 µm sections were cut using a vibratome and collected in sterile culture dishes containing PBS. Sections were processed for URP-mRNA in situ hybridization as described above using the same sequences and labelling procedure. After hybridization and washes, sections were then pre-incubated in a solution containing 2% of BSA and 0.1% of Triton X100 diluted in 0.05 M PBS for 1 h at RT. Sections were immersed overnight at 4°C in a solution containing sheep alkaline-phosphatase conjugated anti-digoxigenin F(ab) fragment (1∶5000, Roche, Mannheim, Germany) and monoclonal mouse anti-GnRH (1∶1000, Sternberger Monoclonals Incorporated, Lutherville, Maryland, USA), in order to localize simultaneously URP-mRNA by *in situ* hybridization and GnRH peptide by immunohistochemistry. Afterwards, sections were washed in a buffered solution (0.1 M Tris, pH 7.5, 1 M NaCl, 5 mM MgCl_2_) and incubated for 2 h at RT with biotinylated goat anti-mouse IgG (1∶200, Vector Laboratories, Burlingam, USA) diluted in the buffered solution added with 2% BSA. This incubation was followed by an incubation in a solution containing HRP-conjugated streptavidine diluted 1∶250 for 1 h at RT. After washes, HRP activity was revealed using a solution containing 0.05% DAB (Sigma) and 0.006% H_2_O_2_ in 0.05 M Tris buffer, pH 7.6 for 10 min at RT. Reaction was stopped by immersion in Tris buffer. Finally, sections were processed for ISH signal revelation as described above. Sections were examined on a Zeiss Axiovert 200 M microscope (Göttingen, Germany) equipped with a black and white Axiocam MRm camera.

To analyse the possible co-localization of URP-mRNA and GnRH peptide in light microscopy, we have used the color deconvolution method [22] a plug-in for ImageJ software [23]. This procedure implements stain separation in images. To provide a more accurate stain separation, new vectors have been determined for sections revealed with DAB (GnRH immunochemistry) and NBT/BCIP staining (URP mRNA) in the same experimental conditions as the double immunostaining.

### Primary culture of anterior pituitary cells

Anterior pituitary glands were removed from Wistar rats and cells were enzymatically dispersed using the trypsin dissociation procedure, as previously described [24]. Briefly, pituitaries were cut into small pieces and digested with 5 mg/ml trypsin. Dispersed cells were plated at a density of 2×10^6^ cells in 12-well plates in Ham's F-10 medium (Sigma) containing 10% fetal calf serum (PAA Laboratories, France) and antibiotics (100 U/ml penicillin and 100 µg/ml streptomycin). Pituitary cells were incubated for 3 days at 37°C in a humidified atmosphere with 5% CO_2_ before treatments. After an overnight incubation in serum-free Ham's F-10, cells were treated for 4 or 18 h with freshly reconstituted URP (1; 10; 100 or 1000 nM) alone or in combination with 0.1 nM GnRH agonist [D-Trp^6^]GnRH (Triptorelin, GnRHa). After stimulation, the medium was collected and stored at −20°C for determination of LH or FSH release. All the experiments were performed in triplicate.

### LH and FSH assays

LH and FSH concentrations were measured by radioimmunoassay in the culture medium of pituitary cells using a commercial radioimmunoassay kits (Immunodiagnostic Systems, France) that displays a sensitivity of 0.14 ng/ml.

## Results

### URP expression in the rat hypothalamus

**Figure 1:**
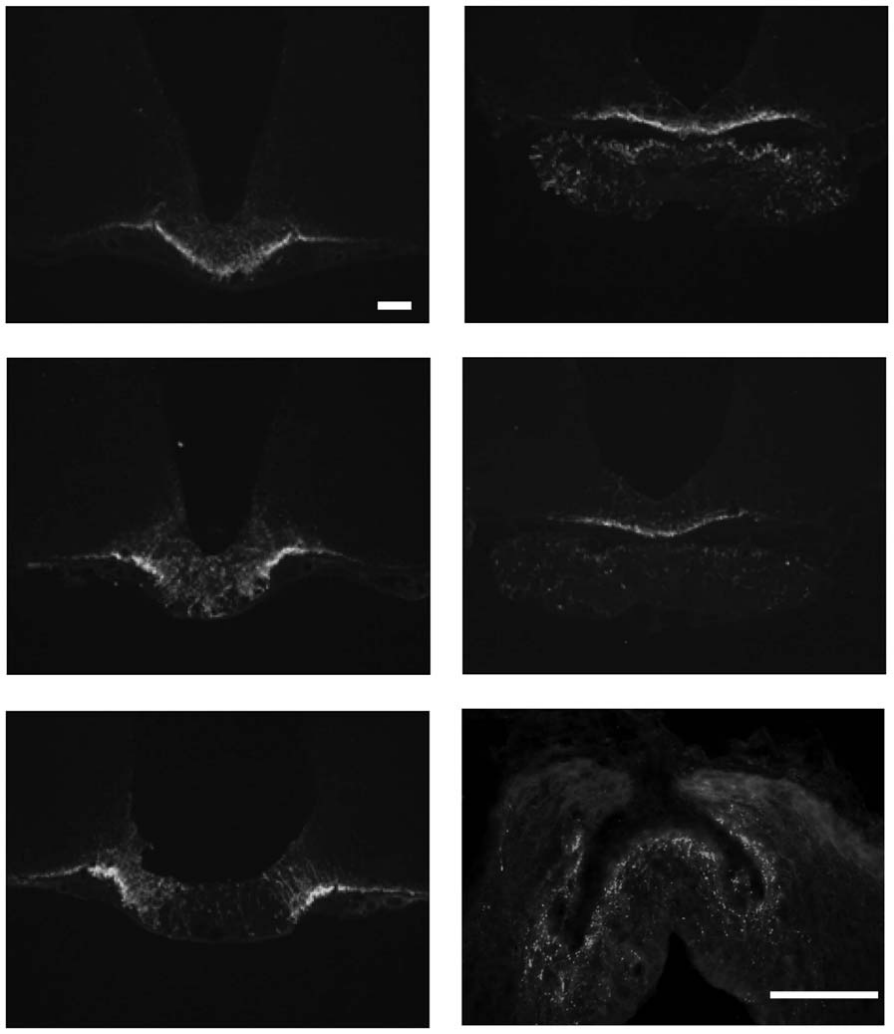
URP distribution in the rat hypothalamus. Frontal (A–E) and horizontal (F) sections of rat hypothalamus, including respectively the median eminence (A–C) up to the infundibular stalk of the pituitary gland (D–E) and the *organum vasculosum laminae terminalis* (F), after treatment with anti-URP antibodies revealed by immunofluorescence. Labelled fibres of variable intensity are detected at all levels of the ME, especially in the outer palissade layer. In OVLT, immunofluorescent fibres are in close contact with the vessels (F). Bars represent 500 µm (A–E) and 100 µm (F). Asterisk: third ventricle.

URP-immunoreactive axons and terminals detected in hypothalamus were exclusively observed in the median eminence (ME) and in the *organum vasculosum laminae terminalis* (OVLT) (Fig. 1). No URP-immunoreactive processes were found outside the hypothalamus. In the basal hypothalamus, the highest concentrations of labelled axons and terminals were found in the external layer of the ME. In the more rostral part of the ME, the entire external layer was immunoreactive (Fig. 1A), but in more caudal parts the labelled fibres were confined to the lateral aspects, with only few fibres in the medial parts (Fig. 1 B–C). In the posterior basal hypothalamus, labelled fibres were less abundant and occurred in the proximal part of the pituitary stalk (Fig. 1D), all around the structure, before disappearing at the level of the posthypophysis (Fig. 1E). The basal surface at this level of the brain was also covered by URP-positive varicose fibres (Fig. 1D). Finally, a dense plexus of URP-positive fibres was present in the OVLT close to the capillary network (Fig. 1F).

**Figure 2:**
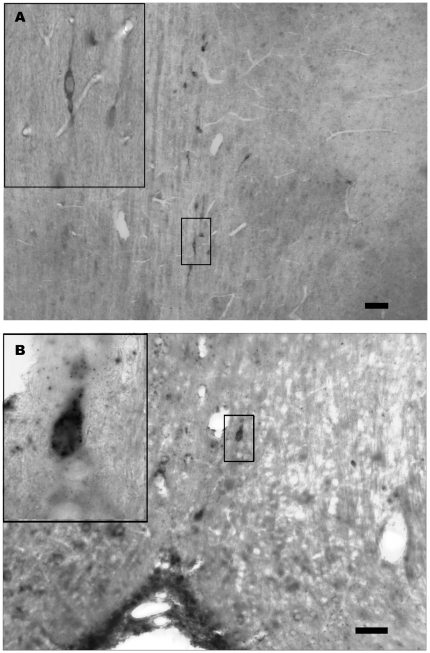
URP expression in the rat hypothalamus. Frontal sections of rat brain after treatment with anti-URP antibodies revealed by immunoperoxidase (A) and after *in situ* hybridization of rat URP-mRNA with Dig-labelled probes (B). Intensely reactive neuronal cell bodies with short labelled process are observed in medial septal nucleus (A) and in the vertical limb of the diagonal band of Broca. Bars represent 100 µm.

In the anterior hypothalamus, most URP-immunoreactive cell bodies were located rostrally (Fig. 2). They were dispersed in the medial septal nucleus and in different nuclei of the diagonal band of Broca. Frequently, they were found in close apposition to blood vessels. The labelled cells were generally small (10–15 µm) and bipolar or unipolar fusiform neurones (Fig. 2A). Majority of cells displayed only one prominent process oriented dorso-ventrally. The peroxidase immunohistochemical studies confirmed the immunofluorescence results illustrated in Fig. 1 by showing the same population of labelled fibres with respect to their number and localization (Fig. 2A). In all experiments, no immunodetection was observed when primary antisera were omitted. Preincubation of the URP-antiserum with rat URP resulted in a complete loss of immunoreaction. In addition, preadsorption of the diluted immune serum by synthetic mammalian GnRH at concentrations of 10^−9^, 10^−8^ and 10^−7^ M did not modify the pattern of immunoreactive fibers in the external zone of the median eminence.

Adjacent sections used for ISH with URP mRNA probes showed a few cell bodies in the medial septal nucleus and in the vertical limb of the diagonal band of Broca which exhibited variable labelling (Fig. 2B). All positive cell bodies displayed the same localization and neuronal characteristics as observed using immunoperoxidase detection of URP (Fig. 2A). No signal was observed on control sections hybridized with sense probes and with an excess of unlabelled antisense probes.

### Co-localization of URP and GnRH

**Figure 3:**
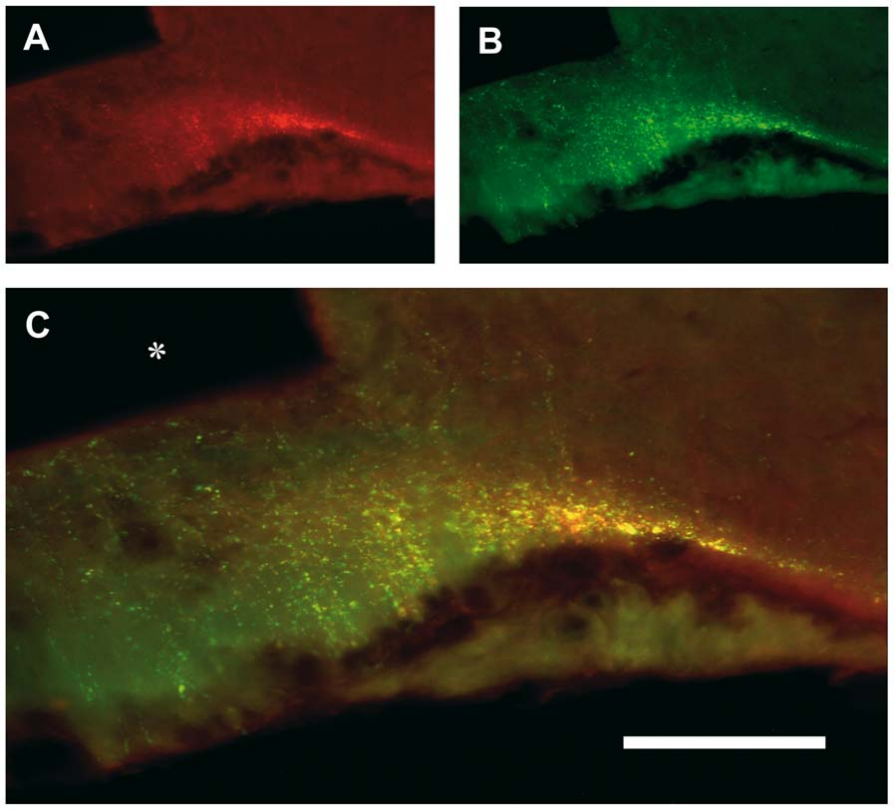
Co-localization of URP and GnRH by double immunohistochemistry. Detail of a frontal section of the median eminence double-immunolabelled for URP and GnRH. URP immunopositive fibres (A) are red while GnRH-immunoreactive fibres (B) are green. Double labelled fibres (C) appear as yellow/orange on the merged projection of twenty successive 1 µm-thick optical sections. Bar represents 500 µm. Asterisk: third ventricle.

Given that URP-positive cell bodies were observed in some hypothalamic areas known to contain GnRH neurones and their processes, we looked for a co-localization of URP and GnRH. Using double immunohistochemistry, we showed that numerous URP-immunoreactive fibres were positive for GnRH peptide in the median eminence, whereas only a subpopulation of GnRH positive fibres was URP-immunopositive (Fig. 3C). Preincubation both of the URP-antiserum with mammalian GnRH peptide or GnRH-antiserum with rat URP synthetic peptide did not reduce the intensity of immunostainings.

**Figure 4:**
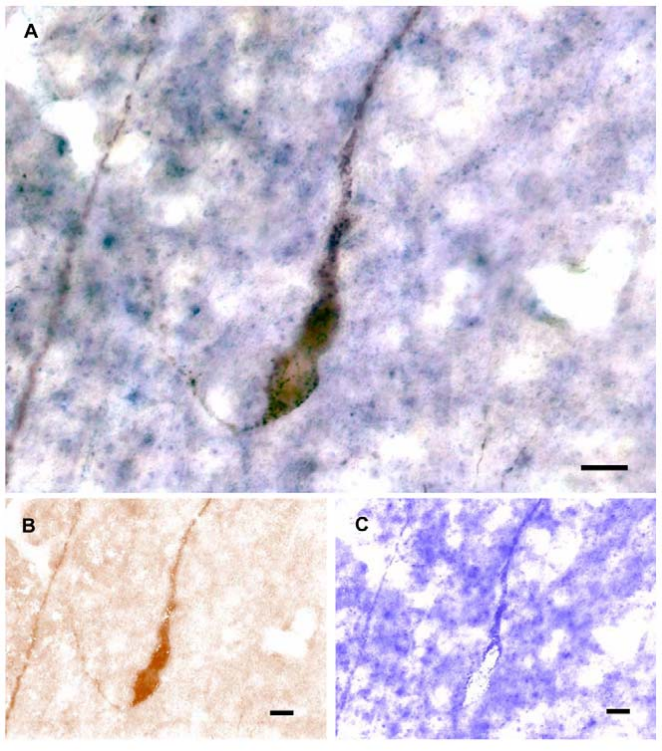
Co-localization of URP mRNA and GnRH peptide. Stain separation by color deconvulation method. Neurones are stained for GnRH by immunohistochemistry with DAB precipitate, URP mRNA is detected simultaneously by in situ hybridization with NBT/BCIP precipitate (A). Stain separation for DAB (B) and NBT/BCIP (C) respectively. Bars represent 10 µm.

Using GnRH immunohistochemistry combined with URP mRNA in situ hybridization, we showed that cell bodies containing both GnRH peptide and URP-mRNA were detected in the anterior hypothalamus, in the same areas where URP-positive neurones were observed using immunofluorescence or immunoperoxidase as described above (Fig. 4A). Using a color deconvolution method, DAB staining revealing GnRH immunodetection (Fig. 4B) and NBT/BCIP staining revealing URP mRNA (Fig. 4C) could be separated in two images. GnRH peptide was located in the whole neurones, soma and processes (Fig. 4B), whereas URP-mRNA was detected mainly in processes and to a lesser extent in the soma near processes (Fig. 4C).

### Subcellular localisation of URP in the rat hypothalamus

To gain further information about URP localization in hypothalamic neurones, we further performed electron microscopic immunocytochemistry.

**Figure 5:**
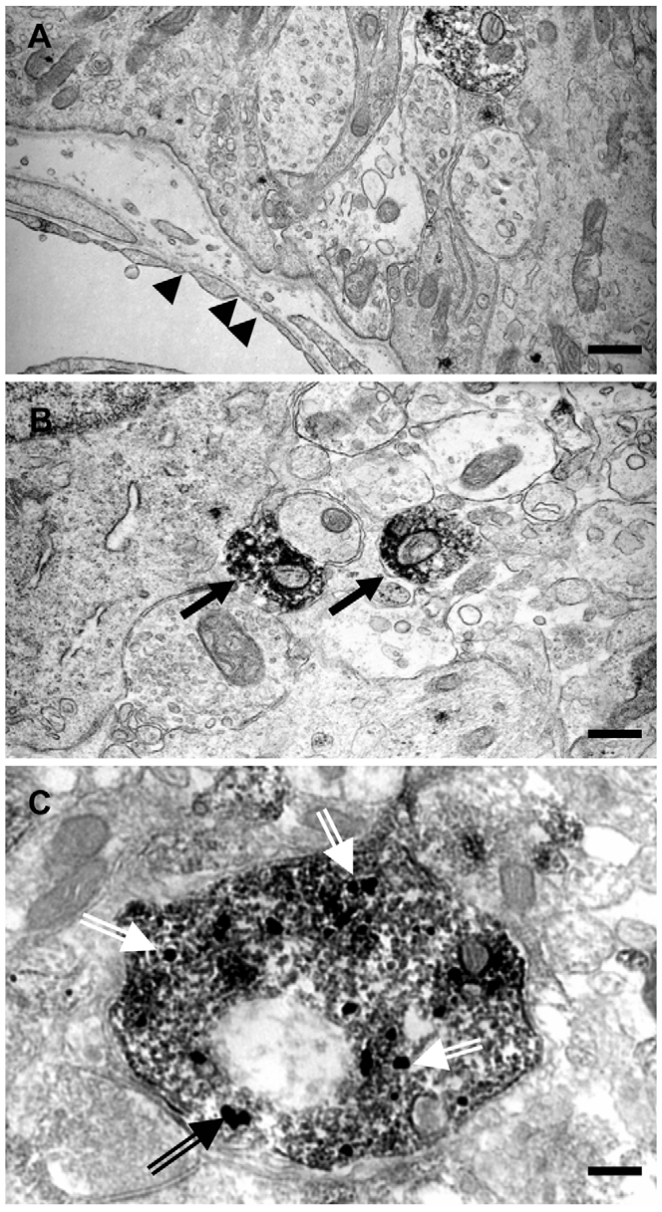
Subcellular co-localization of URP and GnRH. Two ultrathin sections of a vibratome slice of rat median eminence after treatment with antibodies against URP revealed by immunoperoxidase (A and B). The DAB-labelling is observed on fibres (arrows) of the ME. Co-localisation of URP and GnRH peptides in the same processes (C): colloidal gold particles intensified by silver exhibit GnRH peptide (double arrow) whereas DAB precipitate signs the presence of URP (asterisk). Bar represents 0.5 µm (A, B) and 0.25 µm (C). Arrow head: fenestrated capillary. T: tanycyte process.

At ultrastructural level, URP peptide, as revealed by DAB labelling, was observed on process sections, in the axoplasm, in both the internal and external zones of ME. In the inner lateral layer, labelled processes were sometimes very thin. Swellings were in close contact with unlabelled fibres or occasionally cell bodies (Fig. 5A). In the outer layer, immunopositive processes contacted unlabelled axonal sections or tanycyte processes. They were in the vicinity of fenestrated capillaries of the pituitary portal system but never directly in contact with them (Fig. 5B).

In order to confirm at the ultrastructural level the co-localization of URP and GnRH, we next performed a double immunodetection of both peptides. Colloidal gold particles intensified by silver exhibited GnRH peptide whereas DAB precipitate signed the presence of URP. This procedure allowed us to demonstrate that URP and GnRH peptides were co-localized in a same process (Fig. 5C).

### Effect of URP on basal or GnRH-dependant gonadotrophin release in cultured pituitary cells

**Figure 6:**
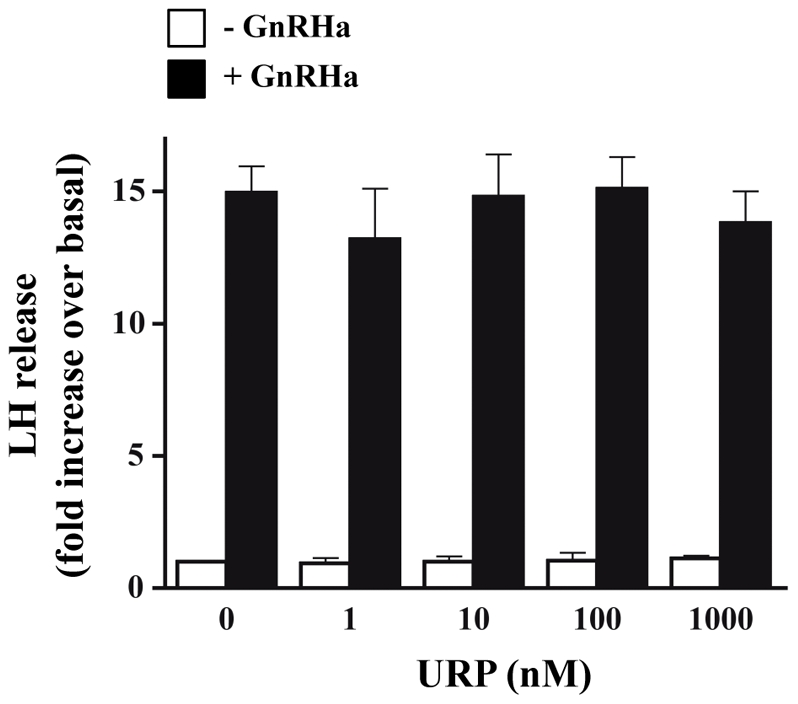
Effect of URP on basal or GnRH-dependant gonadotrophin release in cultured pituitary cells. URP does not influence basal or GnRH-induced LH release. Rat anterior pituitary cells were cultured for 18 h with increasing concentrations (1 to 1000 nM) of URP in combination or not with 0.1 nM of the GnRH agonist Triptorelin (GnRHa). Control cells were treated or not with 0.1 nM GnRHa for the same period of time. LH release into the incubation medium was determined by radioimmunoassay as indicated in *Materials and Methods*. Results are expressed in fold increase over basal LH release in unstimulated cells and are the mean ± SEM of three independent experiments.

Our results demonstrated that URP was co-expressed within GnRH neurones and GnRH-containing processes in the median eminence and prompted us to evaluate whether URP could affect gonadotrophin release. We thus evaluated URP effects *in vitro* on cultured rat anterior pituitary cells on basal or GnRH-dependant LH and FSH release. Fig. 6 shows that treatment of pituitary cells with increasing concentrations of URP (1 to 1000 nM) for 18 h did not interfere with basal LH release (Fig. 6). The GnRH agonist Triptorelin (0.1 nM) efficiently stimulated LH release (15 fold increase over basal), as expected. Co-treatment of cells with increasing concentrations of URP did not significantly affect GnRH-induced LH release (Fig. 6). The same results were obtained for FSH release (data not shown) and also using shorter treatments with URP (4 h; data not shown).

These data indicate that URP did not affect the basal or the GnRH-induced gonadotrophin release. Using a culture of rat anterior pituitary cells, a 15-fold increase in LH release was observed when the GnRH agonist was added during 18 h in the culture medium and in absence of URP, as attempted. However, increasing concentrations (1 to 1000 nM) of URP in combination or not with GnRHa, did not influence LH release from pituitary cells in the culture medium.

## Discussion

Actually, very few data concerning the localization of Urotensin II-Related Peptide (URP) in mammal brain is available, and its physiologic role in the central nervous system remains unknown. In a preliminary work performed with male mouse, we have postulated that URP could be a potential novel hypothalamic neuroendocrine peptide in mammals that is co-expressed in GnRH neurones, suggesting possible interaction between these two peptides for regulating gonadotropic function [12]. Then, presence of URP mRNA has been described in sparse cells located in the vertical limb of the diagonal band nucleus of mouse brain [25]. We have used several different molecular histological and cytological procedures to show the presence of URP and its corresponding mRNA within hypothalamic GnRH neurones of male rat. These current experiments are the first cellular localization of URP in the rat hypothalamus. URP and its corresponding mRNA have been detected in GnRH cells located in the medial septal nucleus and in the diagonal band of Broca. In these cerebral areas, URP mRNA has been detected not only in soma but also in processes. Such subcellular localization of mRNA has been already described in some other systems such as the vasopressin and oxytocin hypothalamic neurone system [26], [27]. The similar pattern of URP peptide and its mRNA localization indicate that the cellular machinery, from transcription of the mRNA to maturation of the peptide, could occur in the same neuronal compartment. URP-immunoreactive processes have been observed in the neurohemal areas of the circumventricular organs OVLT and median eminence. No URP-immunoreactive process has been found outside the hypothalamus. In the median eminence, URP-containing fibres were positive for GnRH peptide, whereas only a subpopulation of GnRH positive fibres is URP-immunopositive. Thus, URP is expressed only in a subpopulation of neuroendocrine GnRH neurones. We precise at the ultrastructural level using electron microscope double immunocytochemistry, the co-localization of URP and GnRH in the same processes located in the mediane eminence.

The dense accumulation of both URP and GnRH in nerve processes in the external zone of the median eminence strongly suggests that URP is important in the neuroendocrine GnRH system controlling the reproductive function. Our functional study shows that URP is unable to increase basal or GnRH-induced LH and FSH release in cultured pituitary cells. So, with our experimental procedure, URP would not interact with GnRH in a synergistic manner at the level of the antehypophysis, but we cannot however exclude totally a role of URP on the basal or GnRH-dependant gonadotrophin release. Recent RT-PCR studies support this discrepancy ∶ authors highlighted that mouse pituitary gland did not show significant expression of URP receptor mRNAs whereas significant amounts were detected in hypothalamus and several structures of the genitourinary tract [25]. In contrast, in rat, URP receptor mRNA was detected by RT-PCR in the hypophysis and in some circumventricular organs such as area postrema and choroid plexus, but no data were mentioned about median eminence [28]. To gain further information, the presence or absence of URP receptors must be elucidated at the cellular and subcellular levels in the hypophysis and the median eminence.

Taking together these data, we can hypothesize that URP would modulate the secretion of GnRH from nerve terminals in the median eminence, as it has been described for the co-expressed neuropeptide galanin [9], [29], [30]. The mechanisms underlying the regulation of GnRH neurones by galanin remain still unknown. The presence of URP mRNA in processes of GnRH neurones could have several physiological roles and could lead to local release of URP. This local release could have consequences on autocrine/paracrine cellular interactions involving axons of the GnRH neurones, astrocytes, tanycytes (specialized unciliated ependymal cells lining the floor of the third ventricle), and endothelial cell of the capillary plexus. The concept that, according to the physiological conditions, the modifications of cellular interactions leading to a structural plasticity would be responsible for a modification of function, is extending in the GnRH system (see for review [8]). To analyse further this hypothesize, expression of URP in the neuroendocrine GnRH system should be evaluated throughout the oestrous cycle of the female rat.
